# Impact of Lymph Node Ratio on the Survival of Patients with Hypopharyngeal Squamous Cell Carcinoma: A Population-Based Analysis

**DOI:** 10.1371/journal.pone.0056613

**Published:** 2013-02-19

**Authors:** Yu-Long Wang, Shou-Hao Feng, Ji Zhu, Guo-Pei Zhu, Duan-Shu Li, Yu Wang, Yong-Xue Zhu, Guo-Hua Sun, Qing-Hai Ji

**Affiliations:** 1 Department of Head & Neck Surgery, Cancer Hospital, Fudan University, Shanghai, People’s Republic of China; 2 Department of Oncology, Shanghai Medical College, Fudan University, Shanghai, People’s Republic of China; 3 Department of Radiation Oncology, Cancer Hospital, Fudan University, Shanghai, People’s Republic of China; 4 Department of Clinical Statistics, Cancer Hospital, Fudan University, Shanghai, People’s Republic of China; Shanghai Jiao Tong University School of Medicine, China

## Abstract

**Objective:**

To analyze the impact of the lymph node ratio (LNR, ratio of metastatic to examined nodes) on the prognosis of hypopharyngeal cancer patients.

**Methods:**

SEER (Surveillance, Epidemiology and End Results)-registered hypopharyngeal cancer patients with lymph node metastasis were evaluated using multivariate Cox regression analysis to identify the prognostic role of the LNR. The categorical LNR was compared with the continuous LNR and pN classifications to predict cause-specific survival (CSS) and overall survival (OS) rates of hypopharyngeal cancer patients.

**Results:**

Multivariate analysis of 916 pN+ hypopharyngeal cancer cases identified race, primary site, radiation sequence, T classification, N classification, M classification, the number of regional lymph nodes examined, the continuous LNR (Hazard ratio 2.415, 95% CI 1.707–3.416, P<0.001) and age as prognostic variables that were associated with CSS in hypopharyngeal cancer. The categorical LNR showed a higher C-index and lower Akaike information criterion (AIC) value than the continuous LNR. When patients (n = 1152) were classified into four risk groups according to LNR, R0 (LNR = 0), R1 (LNR ≤0.05), R2 (LNR 0.05–0.30) and R3 (LNR >0.30), the Cox regression model for CSS and OS using the R classification had a higher C-index value and lower AIC value than the model using the pN classification. Significant improvements in both CSS and OS were found for R2 and R3 patients with postoperative radiotherapy.

**Conclusions:**

LNR is a significant prognostic factor for the survival of hypopharyngeal cancer patients. Using the cutoff points 0.05/0.30, the R classification was more accurate than the pN classification in predicting survival and can be used to select high risk patients for postoperative treatment.

## Introduction

Hypopharyngeal cancer accounts for 2–6% of head neck cancers. [1], [2] The prognosis of patients with cancer of the hypopharynx can be poor despite aggressive combined modality treatment. [3] Traditionally, laryngopharyngectomy with reconstruction of the pharynx has been the preferred initial treatment modality for hypopharyngeal cancers. In an attempt to limit the morbidity of surgical therapy, nonsurgical treatments have gained popularity. [2] While for selected patients with early T classification hypopharyngeal cancer, transoral laser microsurgery in combination with neck dissection and postoperative radiotherapy shows results comparable to those of open surgical procedures and radiotherapy, morbidity and complication rates tend to be lower. [4] For patients with a T4a classification (tumor invasion of thyroid/cricoid cartilage, hyoid bone, thyroid gland or the central compartment of soft tissue), surgery plus neck dissection followed by adjuvant chemotherapy/radiotherapy or radiotherapy has been preferred in accordance to the NCCN guidelines. [3]

When primary surgery is the selected management path for resectable hypopharyngeal cancer cases, postoperative chemotherapy/radiotherapy is recommended (level I evidence) for the adverse pathologic features of extracapsular nodal spread and/or a positive mucosal margin. [3], [5] For other risk features, clinical judgment should be used when deciding to use radiotherapy alone or when considering including chemotherapy with radiotherapy. A better postoperative staging system for hypopharyngeal cancer would be helpful in selecting suitable patients for more intensified postoperative therapy and in the design of clinical trials. The lymph node ratio (LNR), defined as the number of involved nodes divided by the number of lymph nodes examined, was found to improve prognostic information in breast cancer, gastric cancer, colorectal cancer, melanoma and others. [6], [7], [8], [9], [10], [11], [12] Approximately 60% to 80% of patients with hypopharyngeal cancer have locally advanced disease with spread to regional nodes at diagnosis. [13] However, there have been no previous studies on the impact of the LNR on predicting the prognosis of hypopharyngeal cancer.

In the current study, the prognostic role of LNR was analyzed in SEER (Surveillance, Epidemiology and End Results)-registered hypopharyngeal cancer patients with lymph node metastasis. In addition, the cutoff points for the LNR in defining patients as high, medium or low risk groups were identified. The predictive accuracy for survival of the categorical LNR was also compared with the pN classification of hypopharyngeal cancer.

## Materials and Methods

### Patients

The SEER Cancer Statistics Review (http://seer.cancer.gov/), a report on the most recent cancer incidence, mortality, survival, prevalence and lifetime risk statistics, is published annually by the Data Analysis and Interpretation Branch of the National Cancer Institute, MD, USA. [14] SEER collects and publishes these statistics from population-based registries covering 26% of the US population. The 17 SEER registries routinely collect data on patient demographics, primary tumor site, tumor morphology, extent of disease, first course of treatment and active follow-up for vital status. [14] The SEER set has been widely used for the analysis of LNR staging of breast, colon, gastric and other cancers. [11], [15], [16].

Cases of hypopharyngeal carcinoma from 1988 to 2008 were extracted from the SEER database (SEER*Stat 7.0.5) according to the Site Recode classifications. The data were standardized according to the schema of the International Classification of Disease for Oncology. Cancers were limited to the hypopharynx, which were defined as the pyriform sinus (C12.9), postcricoid region (C13.0), hypopharyngeal aryepiglottic fold (C13.1), posterior wall of the hypopharynx (C13.2), overlapping lesion of the hypopharynx (C13.8) and hypopharynx, NOS (C13.9). Histology was limited to squamous cell carcinoma (histology recode - broad groupings 8050–8090). Only hypopharyngeal carcinomas as a single primary tumor or the first diagnosed cancer type of two or more primary tumors were included in current study due to the available information for cause specific survival analysis in SEER database. The LNR was calculated as the number of positive regional nodes (1988+) divided by the number of regional nodes examined (1988+). The cases with disconcordant N classification information and the number of positive regional lymph nodes recorded in SEER database were rejected (N = 116). Finally, a total of 1152 cases of hypopharyngeal carcinoma were collected as the analysis set, and among them 916 cases had pathological lymph node involvement (pN+). This study was based on public use de-identified data from the U.S. SEER database and did not include interaction with human subjects or use personal identifying information. The study did not require informed consent and was approved by the Review Board of Fudan Univesity Shanghai Cancer Center, Shanghai, China. The authors, however, obtained Limited-Use Data Agreements from SEER.

### Statistical Analysis

The categorical and continuous variables of hypopharyngeal cancer cases were retrieved from the SEER database and rechecked. TNM staging information was restaged according to the 2010 American Joint Committee on Cancer (AJCC) staging system, and cases with insufficient classification were defined as X. The CSS (SEER cause-specific survival, http://seer.cancer.gov/causespecific/) was a net survival measure representing survival of a specified cause of death in the absence of other causes of death. The ‘SEER cause-specific death classification’ variable is used to obtain cancer-specific survival probability for a given cohort of cancer patients. Deaths attributed to the cancer of interest are treated as events and deaths from other causes are treated as censored observation. [14] The overall survival (OS) was calculated based on the Vital status recode. [14].

The analysis was carried out in three stages. First, we evaluated the prognostic value of LNR as a continuous variable, adjusting for other covariates associated with CSS in 916 pN+ hypopharyngeal cancer cases. In the second stage, we proceeded to determine the most appropriate cutoff points for categorizing the LNR into high, medium and low risk groups, and compared the predictive accuracy for survival between the categorical LNR and the continuous LNR. The fist pair of cutoff points was identified by means of tertiles of continuous LNR. The second pair of cutoff points were analyzed using the X-tile program (http://www.tissuearray.org/rimmlab/), which identified the cutoff with the minimum *P* values from log-rank χ^2^ statistics for the categorical LNR in terms of survival. [8], [17] The third pair of cutoff points were calculated according to Akaike information criterion (AIC) selection for the multivariate Cox regression model. [7] Using R software with the MASS, Boot and Survival package, the possible pair of cutoff points with the maximum likelihood associated with the multivariate Cox regression model, which ranged from 0.05 to 0.95 at intervals of 0.05, was identified as the third pair of cutoff points. Patients with hypopharyngeal cancer (N = 1152) were classified into four categories (R0-3) according to the identified LNR cutoff points. The prognostic significance of categorical LNR (R classification) was compared with the pN classification.

Survival curves were plotted and survival rates were calculated using the Kaplan-Meier method. Statistical comparisons of different factors related to mortality were undertaken using the Log-rank χ^2^ test. In multivariate analysis, forward stepwise regression analysis was carried out using a Cox proportional hazards model. Harrell’s concordance index (C-index) and AIC values related to the Cox regression model were analyzed to compare the predictive ability of the continuous LNR and categorical LNR for the identification of the optimal cutoff points for the LNR, and the R and pN classifications. [18], [19] A higher C-index value and a lower AIC value indicated a more desirable model for predicting outcome. A *P* value of 0.05 was considered statistically significant. All statistical analyses were carried out using SPSS software version 17.0 (SPSS Inc., Chicago, IL) and R2.14.0 software with packages. [20].

## Results

### Lymph Node Ratio as a Prognostic Factor for Survival in Hypopharyngeal Cancer

The clinical characteristics of the 916 SEER patients with pN+ hypopharyngeal cancer are shown in Table 1. The median age of patients was 61 years (range 35–93 years). The median number of lymph nodes examined, positive lymph nodes and lymph node ratio were 25 (range 1–90), 2 (range 1–90) and 0.14 (range 0.01–1.00), respectively. The median follow-up time was 23 months (range, 0–238 months). There were 495 cases with cancer caused death, 181 cases with non-cancer caused death, 238 alive cases and 2 cases lost during follow-up at the end of follow-up. Kaplan-Meier estimates of 1-, 3-, 5- and 10-year CSS were 76.3%, 51.0%, 42.3% and 33.6%, respectively, and estimates of 1-, 3-, 5- and 10-year OS were 76.3%, 43.9%, 30.9% and 18.7%, respectively. The case number and 5-year CSS of patients diagnosed at 1988–1994, 1995–2001, 2002–2008 were 282, 298, 336 and 42.1%, 43.1%, 41.4%, respectively. There was no changes of the survival for pN+ hypopharyngeal cancer cases diagnosed at different period (Log-rank χ^2^ 0.156, *P* = 0.925). The univariate analysis showed that race, primary site, radiation sequence, cancer directed surgery, T classification, N classification and M classification were all associated with CSS in 916 pN+ hypopharyngeal cancer cases. When these factors were assessed using multivariate Cox regression analysis, we found that race, primary site, radiation sequence, T classification, N classification, M classification, the number of regional lymph nodes examined, continuous LNR and age were all independent variables (Table 2).

**Table 1:** Clinicopathological characteristics and Kaplan-Meier cause specific survival (CSS) analysis of SEER hypopharyngeal cancer cases with pathological lymph node involvement.

Variables	No.	5-year CSS	Log-rank χ^2^	*P* value
Race			29.840	<0.001
White	696	46.2%		
Black	155	26.7%		
Other	65	37.5%		
Gender			0.303	0.582
Male	750	41.7%		
Female	166	44.8%		
Year of diagnosis			0.156	0.925
1988–1994	282	42.1%		
1995–2001	298	43.1%		
2002–2008	336	41.4%		
Primary site			17.637	0.003
Pyriform sinus	664	43.2%		
Postcricoid region	20	28.7%		
Aryepiglottic fold	25	63.4%		
Posterior wall	36	42.0%		
Overlapping lesion	24	13.8%		
Hypopharynx, NOS	147	42.1%		
Histological grade			3.230	0.520
I	28	36.5%		
II	370	44.6%		
III	406	40.8%		
IV	16	0		
Unknown	96	39.9%		
Surgery of primary site			1.317	0.725
None	216	40.0%		
Local excision	92	48.4%		
Pharyngectomy	595	42.4%		
Unknown	13	0		
Radiation sequence			25.588	<0.001
No radiation	226	34.5%		
Pre-operative	50	35.1%		
Post-operative	620	46.1%		
Other	20	0		
Cancer directed surgery			7.519	0.006
Yes	747	43.2%		
Other	169	38.9%		
T stage			38.547	<0.001
T1	81	65.9%		
T2	152	56.1%		
T3	144	39.6%		
T4	376	33.2%		
Tx	163	41.8%		
N stage			15.483	<0.001
N1	194	51.4%		
N2	653	41.5%		
N3	69	23.0%		
M stage			46.848	<0.001
M0	871	43.8%		
M1	31	5.4%		
Mx	14	24.2%		

**Table 2:** Multivariate analysis of the LNR and covariates associated with SEER cancer cause-specific survival of hypopharyngeal cancer cases with lymph node metastasis.

Variables	HR (95% CI)	*P* value
Race (White as reference)		
Black	1.678(1.334–2.112)	<0.001
Other	1.392(0.999–1.940)	0.051
Primary site (Pyriform sinus as reference)		
Postcricoid region	1.088(0.619–1.911)	0.769
Aryepiglottic fold	0.664(0.338–1.305)	0.235
Posterior wall	2.327(1.459–3.711)	<0.001
Overlapping lesion	2.140(1.327–3.453)	0.002
Hypopharynx, NOS	1.206(0.935–1.555)	0.149
Radiation sequence (no radiation as reference)		
Pre-operative	0.976(0.642–1.482)	0.908
Post-operative	0.643(0.512–0.807)	<0.001
Other	1.060(0.580–1.936)	0.850
T classification (T1 as reference)		
T2	1.441(0.906–2.292)	0.122
T3	2.504(1.597–3.925)	<0.001
T4	2.965(1.954–4.497)	<0.001
Tx	1.671(1.055–2.648)	0.029
N classification (N1 as reference)		
N2	1.352(1.064–1.717)	0.014
N3	2.224(1.544–3.202)	<0.001
M classification (M0 as reference)		
M1	4.195(2.679–6.569)	<0.001
MX	1.289(0.636–2.613)	0.482
No. of regional lymph node examined	1.009(1.003–1.014)	0.002
LNR as continuous variable	2.415(1.707–3.416)	<0.001
Age	1.017(1.007–3.416)	<0.001

### Identification of LNR Cutoff Points

The complexity of the underlying computations for obtaining the hazard ratios for the continuous LNR ensures that the immediately readable hazard ratios for the categorized LNR are preferred in daily clinical practice. To stratify the patients with lymph node metastasis as high, medium and low risk groups associated with CSS, the upper and lower tertiles of continuous LNR that corresponded to 0.09 and 0.27 were defined as the first pair of cutoff points. The X-tile, which can control the inflated type I error problem and minimize the loss of information due to multiple testing through cross-validation, identified 0.08/0.33 as the second pair of cutoff points. [8], [17] After recomputing the likelihood associated with all pairs of LNR cutoffs (dividing patients into three risk groups) using the multivariate regression model (Table 2), 0.05/0.30 was identified as the third pair of cutoff points with the maximum likelihood associated with the Cox regression model. The SEER cases with lymph node metastasis were stratified as high, medium and low risk groups according to the three pairs of cutoff points identified above. The case numbers, the 5-year CSS and 5-year OS of the different risk groups were summarized in Table 3. To compare the predictive ability of the categorical LNR and the continuous LNR, the C-index and AIC value of the Cox regression model (Table 2) with substitution of the continuous LNR with the categorical LNR were calculated. As listed in Table 3, all three pairs of the categorical LNRs showed superior predictive ability to that of the continuous LNR, with the lower AIC value and higher C-index value associated with the Cox regression model. For the sake of convenience in clinical interpretation and application, the cutoff points 0.05/0.30 with the lowest AIC values were used for further analysis.

**Table 3:** Univariate and multivariate analysis of the categorical and continuous LNR with cause-specific survival (CSS) and overall survival (OS) of SEER hypopharyngeal cancer patients with lymph node metastasis.

LNR classification	No.	5-y CSS(%)	Log-rank χ^2^ †	HR (95% CI) ‡	C-index‡	AIC‡	5-y OS(%)	Log-rank χ^2^ #	HR (95% CIs) *	C-index*	AIC*
Continuous LNR				2.415(1.707–3.416)	0.688	5946.70			2.005(1.483–2.712)	0.668	8001.08
Cutpoints 0.09/0.27			39.326		0.695	5943.40		28.887		0.672	7997.18
R1∶0–0.09	330	51.6		Reference			38.9		Reference		
R2∶0.09–0.27	285	40.9		1.451(1.131–1.861)			30.0		1.328(1.076–1.639)		
R3: >0.27	301	32.6		2.150(1.631–2.845)			22.4		1.855(1.463–2.353)		
Cutpoints 0.08/0.33			35.427		0.698	5937.27		37.160		0.675	7988.71
R1∶0–0.08	301	52.0		Reference			39.5		Reference		
R2∶0.08–0.33	357	41.9		1.523(1.190–1.948)			30.1		1.343(1.090–1.653)		
R3: >0.33	258	30.3		2.488(1.848–3.350)			21.3		2.160(1.672–2.790)		
Cutpoints 0.05/0.30			33.768		0.697	5934.87		33.894		0.674	7988.71
R1∶0–0.05	177	55.6		Reference			43.1		Reference		
R2∶0.05–0.30	458	43.6		1.686(1.262–2.252)			31.6		1.461(1.151–1.856)		
R3: >0.30	281	30.7		2.822(2.011–3.959)			21.3		2.301(1.729–3.062)		

† The *P* value for Log-rank χ^2^ tests of cause specific survival was less than 0.001.

‡ Being calculated using the multivariates Cox regression model for cause specific survival with either continuous or categorical LNR and variates listed at Table 2 as covariates.

# The *P* value for Log-rank χ^2^ tests of overall survival was less than 0.001.

* Being calculated using the multivariates Cox regression model for overall survival with either continuous or categorical LNR and variates listed at Table 2 as covariates.

### Superiority of the R Classification Over the pN Classification in the Prediction of Survival

**Figure 1:**
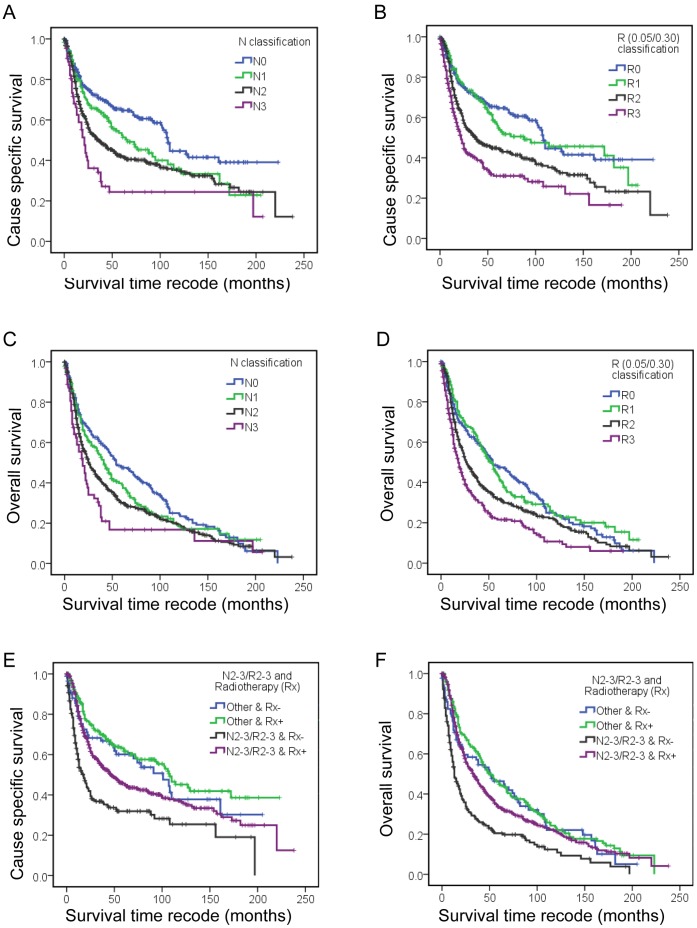
Kaplan-Meier survival estimates for different patient groups. SEER cause-specific survival of the hypopharyngeal cancer cases with different pN classification (A) and R classification (B). Overall survival for different pN classifications (C) and R classifications (D). The SEER cause-specific survival (E) and overall survival (F) differences observed in N2-3/R2-3 group and other hypopharyngeal cancer patients with and without postoperative radiotherapy.

Since the N classification and LNR are both lymph node staging system, the 1152 SEER cases of hypopharyngeal carcinoma were classified as the four risk groups, R0 (LNR, 0), R1 (LNR, 0–0.05), R2 (LNR, 0.05–0.30), R3 (LNR, >0.30) and defined as the R classification to compare with traditional N classification. Univariate analysis (Table 4) and multivariate Cox regression analysis (Table 5) indicated that race, primary site, T classification, N classification, M classification, radiation sequence, cancer directed surgery and age had a significant association with CSS. When substituting the N classification with the R classification, the Cox regression model of CSS for the R classification showed a lower AIC value and higher C-index value, which suggested that Cox regression model with R classification had better predictive ability (Table 5). The Cox regression model for the R classification was also superior to the N classification in predicting OS (Table 5). Table 5 and Figure 1 show the univariate Kaplan-Meier CSS and OS estimates according to risk groups, defined using the pN (Fig. 1A and C) or R classification (Fig. 1B and D). As shown at Figure 1, for the 5-year CSS or OS, both the N and R classifications showed good discriminatory ability among each group. For the 10-year CSS and OS, the survival curves for N1 and N2 crossed at 35% and 19%. The 15-year CSS and OS survival curves for N1, N2 and N3 classifications converged at 23% and 10%, respectively (Table 5). In comparison, the 10-year CSS and OS curves for the R1-3 classification were clearly separated even with a follow-up time exceeding 15 years. For the R classification, similar survival curves were obtained for the R1 (lower LNR) and R0 (no lymph node metastasis) groups (Fig. 1B and D), and the adjusted hazard ratio (95% CIs) of the Cox regression model for the R1 group was 1.134 (0.901–1.439) for CSS and 1.072 (0.850–1.352) for OS, when using R0 as the reference group (Table 5).

**Table 4:** Univariate analysis of clinicopathological variables associated with SEER cause-specific survival (CSS) and overall survival (OS) of hypopharyngeal cancer patients (n = 1152).

Patients’ variables	No.	5-y CSS (%)	Log-rank χ^2^	*P* value	5-y OS(%)	Log-rank χ^2^	*P* value
Race			28.206	<0.001		26.402	<0.001
White	872	50.5			37.1		
Black	198	33.8			23.9		
Other	82	42.1			33.9		
Primary site			18.132	0.003		9.588	0.088
Pyriform sinus	829	48.0			34.8		
Postcricoid region	24	33.4			23.2		
Aryepiglottic fold	38	69.6			52.0		
Posterior wall	48	39.9			27.6		
Overlapping lesion	27	21.6			18.3		
Hypopharynx, NOS	186	48.2			34.1		
T classification			37.299	<0.001		36.991	<0.001
T1	93	68.7			53.9		
T2	203	61.5			47.0		
T3	181	43.0			32.5		
T4	484	39.5			28.1		
Tx	191	44.5			30.4		
N classification			41.026	<0.001		22.437	<0.001
N0	236	64.7			47.3		
N1	194	51.4			38.8		
N2	653	41.5			29.8		
N3	69	23.0			15.8		
M classification			49.844	<0.001		46.822	<0.001
M0	1099	48.7			35.5		
M1	36	12.3			7.0		
Mx	17	30.5			20.2		
Radiation sequence			24.501	<0.001		26.596	<0.001
No radiation	292	40.4			28.3		
Pre-operative	61	40.3			35.8		
Post-operative	776	50.8			36.9		
Other	23	35.9			21.7		
Cancer directed surgery			18.421	<0.001		14.241	<0.001
Yes	970	48.6			35.4		
Other	182	38.5			28.8		

**Table 5:** Different staging systems for SEER cause-specific survival (CSS) and overall survival (OS) of SEER patients with hypopharyngeal cancer.

Staging system	No.	5/10/15-y CSS(%)	log-rank χ^2^ †	HR (95% CI) ‡	C-index‡	AIC‡	5/10/15-y OS(%)	log-rank χ^2#^	HR (95% CIs) *	C-index*	AIC*
N classification			41.026		0.683	7359.73		22.437		0.657	10428.44
N0	236	64.7/45.7/40.3		Reference			47.3/24.5/12.4		Reference		
N1	194	51.4/35.6/22.8		1.406(1.048–1.887)			38.8/19.2/11.2		1.134(0.901–1.439)		
N2	653	41.5/34.1/26.5		1.926(1.523–2.435)			29.8/19.0/9.6		1.515(1.266–1.812)		
N3	69	23.0/23.0/23.0		2.869(1.984–4.148)			15.8/15.8/10.6		1.954(1.422–2.683)		
R classification			61.697		0.686	7352.23		49.499		0.661	10417.85
R0	236	64.7/45.7/40.3		Reference			47.3/24.5/12.4		Reference		
R1∶0–0.05	177	55.6/45.4/41.3		1.256(0.930–1.698)			43.1/24.7/18.1		1.072(0.850–1.352)		
R2∶0.05–0.30	458	43.6/33.3/22.5		1.922(1.507–2.452)			31.6/20.7/8.1		1.470(1.218–1.775)		
R3: >0.30	281	30.7/25.8/16.9		2.497(1.882–3.314)			21.3/11.0/6.4		1.952(1.556–2.448)		

† The *P* value for Log-rank χ^2^ tests of cause specific survival was less than 0.001.

‡ Being calculated using the multivariates Cox regression model for cause specific survival with either N classification or R classification and variates listed at Table 4 as covariates.

# The *P* value for Log-rank χ^2^ tests of overall survival was less than 0.001.

* Being calculated using the multivariates Cox regression model for overall survival with N classification or R classification and variates listed at Table 4 as covariates.

### R Classification as an Index for Selecting High Risk Patients for Postoperative Radiotherapy

To analysis the role of the R classification in the management of hypopharyngeal cancer, the cases with (n = 776) and without (n = 292) postoperative radiotherapy according to the Radiation Sequence with Surgery recorded in SEER database were used for further study. Surgery and postoperative radiotherapy improved the CSS for N2 and N3 cases, whereas increased OS was only observed in N2 patients (Table 6). In the case of the R classification, an improvement in both CSS and OS was found for R2 and R3 patients who had received postoperative radiotherapy. To select high risk patients for postoperative radiotherapy, we defined the patients with a N2/N3 classification (n = 669/1068, 62.6%) and R2/R3 classification (n = 682/1068, 63.9%) as the N2-3/R2-3 group (n = 763/1068, 71.4%), a significant improvement (*P*<0.001) in both the CSS and OS was observed in the N2-3/R2-3 patients who had received postoperative radiotherapy (Table 6; Fig. 1E and F).

**Table 6:** The cause-specific survival (CSS) and overall survival (OS) differences between different N classification or R classification patients with and without postoperative radiotherapy (Rx).

Patients’ variables	No.	5/10-y CSS (%)	Log-rank χ^2^ †	*P* value	5/10-y OS(%)	Log-rank χ^2^ †	*P* value
N classification							
N0 & Rx –	66	60.1/31.7	3.507	0.061	45.5/17.2	3.139	0.076
N0 & Rx +	156	66.7/50.2			48.3/27.2		
N1 & Rx –	72	51.5/34.1	1.360	0.243	33.9/21.0	1.567	0.211
N1 & Rx +	105	53.9/37.5			42.6/17.9		
N2 & Rx –	129	28.6/28.6	24.185	<0.001	18.4/11.7	33.221	<0.001
N2 & Rx +	476	45.7/36.4			33.3/21.0		
N3 & Rx –	25	18.4/18.4	5.591	0.018	17.6/17.6	2.602	0.107
N3 & Rx +	39	28.3/28.3			15.5/15.5		
R classification							
R0 & Rx –	66	60.1/31.7	0.984	0.061	45.5/17.2	3.139	0.076
R0 & Rx +	156	66.7/50.2			48.3/27.2		
R1 & Rx –	34	64.0/64.0	13.402	0.321	50.9/42.0	1.037	0.308
R1 & Rx +	130	53.5/40.8			40.8/19.3		
R2 & Rx –	68	27.4/27.4	8.842	<0.001	13.7/11.8	23.053	<0.001
R2 & Rx +	350	47.9/35.8			35.4/22.9		
R3 & Rx –	124	29.2/17.2	17.632	0.003	20.4/7.9	7.398	0.007
R3 & Rx +	140	32.4/32.4			22.3/12.7		
N+R classification							
Other & Rx –	87	60.1/37.8	1.068	0.301	46.4/22.0	0.547	0.459
Other & Rx +	218	61.7/45.1			45.9/22.7		
N2-3/R2-3 & Rx –	205	31.9/25.4	30.584	<0.001	20.4/12.4	37.565	<0.001
N2-3/R2-3 & Rx +	558	45.7/36.5			33.6/21.1		

† For Log-rank χ^2^ test, postoperative radiotherapy (Rx) was defined as factor, the individual R, N and N+R classification were defined as Stratum.

## Discussion

The hypopharyngeal cancer cases with lymph node metastasis were staged as III/IV according to the AJCC TNM staging system. Approximately 70 to 85% of the patients reported in larger series were diagnosed as having stage III or IV disease at presentation. [3] The management of stages III/IV hypopharyngeal cancer has been relatively unsatisfactory owing to local recurrence and distant metastasis. Optimal therapy has remained a matter of debate, especially with regard to treatment intensity and sequencing. [2] Although treatment strategies of cancer advance tremendously, no significant survival improvements were observed in pN+ hypopharyngeal cancer patients diagnosed at different periods as shown in current results, which was also observed for laryngeal cancer and supposed to be caused by inappropriate patients selecting and lack of better index for guiding postoperative therapy. [21], [22], [23] It is plausible that selecting high risk patients is important in the choice of optimal treatment strategies. Current study clearly identified that the LNR is an important prognostic factor for CSS in hypopharyngeal cancer patients with lymph node metastasis. As compared with the N classification, the R classification using the cutoff points 0.05/0.30 showed better predictive ability for CSS and OS, with a lower AIC value and a higher C-index value. The survival curves for R1, R2 and R3 patients were clearly separate even after a 15-year follow-up period, whereas intersection of the survival curves for N 1–3 patients was found after a 10-year follow-up period. The similar survival estimates for R1 and R0 patients further confirmed the role of LNR in stratifying patients (a lower LNR to select lower risk patients).

Over the past three decades, more attention has been paid to the identification of factors that might help the surgeon to assess the precise risk of failure in individual patients. [20], [24] The best predictive index should be easily assessed and dependent on the target measured. For example in primary radiotherapy, volume of the primary tumor may be a predictive factor. [25], [26] For surgical approaches to organ preservation, anatomic sub-localization and extension of the primary tumor are more relevant factors. In the case of postoperative radiotherapy for hypopharyngeal cancer, adverse features were defined as extracapsular nodal spread, positive margins, pT3 or pT4 primary tumor, N2 or N3 nodal disease, selected pT1-T2, N0-N1, perineural invasion and vascular embolism according to the NCCN guidelines. [3] When the CSS and OS of individual R classification patients, with or without postoperative radiotherapy, were compared in the current study, survival benefits were observed for R2 and R3 patients. Combining the N and R classifications together can also select more high risk patients for postoperative radiotherapy than using the N classification alone (62.6% vs. 71.4%). All these results support the fact that R2 and R3 should be defined as adverse features for the postoperative management of hypopharyngeal cancer. Because information regarding the details of radiotherapy (the radiation techniques, dose delivered and fractions, and use of concurrent chemotherapy, etc) and pathological reports (margin status, extracapsular extension, perineural invasion and vascular embolism, etc) are not available in the SEER database, the definitive role of the R classification in postoperative staging and selecting high risk patients for postoperative concurrent chemoradiotherapy could not be discussed in the current study, and is deserving of further research.

Although we have adjusted for all available patient and tumor characteristics in our analysis, as with all SEER based studies, our study was limited by the information available in the database. The SEER database is dependent on the individual physician reporting on the classification of the patient, tumor and treatment characteristics. In this way, it is representative of national patterns of cancer care and has a large population base; however, it is also subject to inconsistencies. Although the number of lymph nodes examined and the number of positive nodes is information that can easily be reported with accuracy, there are still cases that were rejected for inclusion in the current study because of disconcordant information with regard to the N classification and the number of lymph node metastases. The quality of neck dissection achieved by the surgeon, and the quantity of lymph nodes harvested and examined by the pathologist will substantially change the LNR and the results. On the other hand, patients who have a lower number of lymph nodes resected (a relatively lower quality of operation), which may cause inflation of the LNR and a higher R classification, will be assessed as a high risk group. The LNR is an index not only of disease burden but also of surgical standards. This is the reason why the current study, together with studies on cancer from other sites, showed that the LNR improved the predictive ability of survival relative to the N classification. [8], [9], [16].

In conclusion, our analysis of the SEER database revealed a significant association between the LNR and survival of hypopharyngeal cancer patients. Using the cutoff points 0.05/0.30, the hypopharyngeal cancer patients with lymph node metastasis were classified into R1, R2 and R3 risk groups. The R classification can be used together with the N classification to select high-risk patients for postoperative treatment. However, more and prospective studies were still needed to confirm the prognostic role of LNR for hypophayryngeal cancer and more clinical evidence should be obtained.
